# TRAIL and Ceruloplasmin Inverse Correlation as a Representative Crosstalk between Inflammation and Oxidative Stress

**DOI:** 10.1155/2018/9629537

**Published:** 2018-07-26

**Authors:** Veronica Tisato, Stefania Gallo, Elisabetta Melloni, Claudio Celeghini, Angelina Passaro, Giorgio Zauli, Paola Secchiero, Carlo Bergamini, Alessandro Trentini, Gloria Bonaccorsi, Giuseppe Valacchi, Giovanni Zuliani, Carlo Cervellati

**Affiliations:** ^1^Department of Morphology, Surgery and Experimental Medicine and LTTA Centre, University of Ferrara, Ferrara, Italy; ^2^Department of Medical Science, University of Ferrara, Ferrara, Italy; ^3^Department of Biomedical and Specialist Surgical Sciences, University of Ferrara, Ferrara, Italy; ^4^Department of Life Sciences and Biotechnology, University of Ferrara, Ferrara, Italy; ^5^Department of Animal Sciences, Plants for Human Health Institute, NC State University, NC Research Campus, Kannapolis, NC, USA

## Abstract

**Objective:**

“Oxinflammation” is a recently coined term that defines the deleterious crosstalk between inflammatory and redox systemic processes, which underlie several diseases. Oxinflammation could be latently responsible for the predisposition of certain healthy individuals to disease development. The oxinflammatory pathway has been recently suggested to play a crucial role in regulating the activity of TNF-related apoptosis-inducing ligand (TRAIL), a TNF superfamily member that can mediate multiple signals in physiological and pathological processes. Therefore, we investigated the associations between TRAIL and key players of vascular redox homeostasis.

**Methods:**

We measured circulating TRAIL levels relative to praoxonas-1, lipoprotein phospholipase-A2, and ceruloplasmin levels in a cohort of healthy subjects (*n* = 209).

**Results:**

Multivariate analysis revealed that ceruloplasmin levels were significantly inversely associated with TRAIL levels (*r* = −0.431, *p* < 0.001). The observed association retained statistical significance after adjustment for additional confounding factors. After stratification for high-sensitivity C-reactive protein levels, the inverse association between TRAIL and ceruloplasmin levels remained strong and significant (*r* = −0.508, *p* < 0.001, *R* ^2^ = 0.260) only in the presence of inflammation, confirming the role of inflammation as emerged in *in vitro* experiments where recombinant TRAIL decreased ceruloplasmin expression levels in TNF-treated PBMC cultures.

**Conclusion:**

The results indicated that in an inflammatory milieu, TRAIL downregulates ceruloplasmin expression, highlighting a signaling axis involving TRAIL and ceruloplasmin that are linked via inflammation and providing important insights with potential clinical implications.

## 1. Introduction

Tumor necrosis factor- (TNF-) related apoptosis-inducing ligand (TRAIL) is a pleiotropic protein belonging to the TNF superfamily that can prime multiple intracellular signals. The ability of TRAIL to induce apoptosis in cancer cells has been well characterized. However, further detailed studies are required to elucidate the mechanisms underlying the effects of TRAIL on noncancer cells, which range from proapoptotic to survival/proliferative activities, as well as its role in healthy and pathological settings.

In the last decade, our group and other investigators provided evidence of the role of TRAIL in vascular physiology and pathophysiology. Most of the *in vivo* and clinical/epidemiological studies supported the vasoprotective role of TRAIL [1, 2]. Indeed, the administration of recombinant TRAIL to streptozotocin-induced diabetic APOE knockout mice led to a significant reduction in plaque mass [3]. In addition, deletion of the TRAIL gene was found to be associated with accelerated atherosclerosis in other animal models [4, 5]. Moreover, previous studies reported an inverse correlation between circulating TRAIL and cardiovascular diseases (CVDs) and diabetes [6–8]. On the other hand, low TRAIL levels were associated with poor outcomes in patients with myocardial infraction, coronary syndrome, and heart failure [9, 10].

Nonetheless, further studies are required to investigate the biological mechanisms underlying the protective effects of TRAIL in the vascular system, as well as the downstream and upstream molecular and cellular targets of TRAIL. A hypothesized pathway that has increasingly gained credit involves the complex network of intracellular redox or redox-sensitive signaling pathways [11]. Disruption of the physiological redox homeostasis caused by a permanent toxic challenge (e.g., excessive reactive oxygen species (ROS) production) or inadequate feedback response gives rise to oxidative stress (OxS), an integral aberrant condition characterizing various diseases, particularly CVD [12]. Notably, TRAIL was demonstrated to influence ROS production in *in vitro* models of vascular cells and was found to primarily act by modulating NADPH oxidase (NOX) and endothelial nitric oxide synthase (eNOS) [13], as well as upregulating the expression of the ROS scavenger superoxide dismutase (SOD) [14].

Uncontrolled elevation in extracellular ROS levels leads to the formation of the proatherogenic oxidized LDL (ox-LDL), which in turn significantly contributes to endothelial dysfunction, foam cell formation, and increased platelet aggregation [15]. Iron and copper are two of the main components involved in endogenous reactions that generate ROS. Indeed, iron and copper in their reduced states can lead to the formation of the hydroxyl radical, one of the most reactive ROS, via the Fenton reaction [16]. In this context, ceruloplasmin, the primary plasmatic copper carrier, plays a pivotal role in preventing the formation of extracellular ROS by oxidizing Fe^2+^ to Fe^3+^, thereby promoting the sequestration of iron by apotransferrin [17, 18].

Two enzymes that are bound to circulating lipoproteins, namely, paraoxonase 1 (PON1) and lipoprotein phospholipase A2 (Lp-PLA2), can also effectively counteract LDL oxidation. These two accessory proteins have gained considerable attention in the last two decades because of their strong involvement in atherosclerosis development [19, 20]. PON1 is a widely recognized atheroprotective agent that confers antioxidant and anti-inflammatory protection against HDLs. Individuals with CVD and other diseases were demonstrated to have lower PON1 serum activity compared to controls [19, 21–23]. Several experimental studies clearly showed that PON1 can protect the endothelium from oxidative insults, attenuate the inflammatory responses of macrophages, and mediate cholesterol efflux from the macrophages to HDL [22, 24]. A similar mechanism of action has been suggested for Lp-PLA_2_ [25]. In addition, results of an *in vitro* study suggested that Lp-PLA2 can degrade other phospholipids containing oxidized fatty acyl groups, such as LDLs and cell membranes, to form lysophospholipids, acetate, and oxidized fatty acids [26]. In contrast to PON1 and despite its apparent antioxidant properties, Lp-PLA2 is abundant in nascent atherosclerotic plaques and is involved in multiple stages of atherosclerosis progression, thereby explaining its positive association with increased risk of coronary heart disease [26–28].

Given their relevant roles in physiological and pathological contexts, the expression and secretion of ceruloplasmin, PON1, and Lp-PLA2 from the liver and immune cells are finely modulated. Previous studies have established that TRAIL exerts its protective effects on the vascular system through several mechanisms, including the regulation of the expression of chemokines, cytokines, and redox mediators. In this line, the present study addresses the hypothesis that ceruloplasmin, PON1, and Lp-PLA2 might represent targets of TRAIL. Therefore, we investigated the association between TRAIL levels and the circulating levels of these enzymes in a cohort of apparently healthy individuals.

## 2. Material and Methods

### 2.1. Subjects

Two hundred and fifty elderly Caucasian outpatients (≥65 years) referring to the Internal Medicine (University of Ferrara, Italy) were included in this study. This study conforms to The Code of Ethics of the World Medical Association (Declaration of Helsinki) and was performed according to the guidelines for Good Clinical Practice (European Medicines Agency, http://www.ema.europa.eu). The study was approved by Local Ethics Committee of the involved institution (Province of Ferrara, Italy). Signed informed consent, which was prepared in compliance with local and national ethical guidelines, was obtained from each patient prior to the inclusion into the study. Personal data and medical history were collected by a structured interview from participants.

Individuals included in the study protocol had normal cognitive function as assessed by a standardized battery of cognitive tests as previously described [29] and were independent in the activities of daily living (ADLs). Subjects with diagnosis of severe congestive heart failure (New York Heart Association class III-IV); severe liver, severe kidney disease (defined as subjects with eGFR <30 ml/min/1.73 m^2^); severe chronic obstructive pulmonary, chronic, or acute inflammatory disease (e.g., all acute known infections or rheumatologic conditions); cancer; or taking NSAIDS, antibiotics, or steroids were excluded. Subjects with serum levels of ferritin, folate, or vitamin B_12_ below the lower limit of normal range were excluded from the study to avoid possible cases of evident iron deficiency or vitamin deficiency anemia.

### 2.2. Biochemical Assays

Fresh peripheral blood samples were collected by venipuncture into Vacutainer tubes without anticoagulant after an overnight fast. After 30 minutes of incubation at room temperature, the blood samples were centrifuged at 4.650*g* for 20 min and sera were collected and stored in single-use aliquots at −80°C until analysis. All the assays were performed by UV-VIS spectrophotometric assays in a 96-well plate format by using a Tecan Infinite M200 microplate reader (Tecan Group Ltd., Switzerland).

Briefly, PON1 activity was determined by monitoring the increase in absorbance (412 nm) caused by 4-nitrophenol formation after addition of diluted serum in reaction mixture (1.5 mmol/l paraoxon, 0.9 mol/l NaCl, and 2 mmol/l CaCl2 dissolved in 10 mmol/l Tris-HCl, pH 8) as described [30]. A molar extinction coefficient of 18,000 M^−1^ cm^−1^ was used for the calculation of enzyme activity, expressed in units per liter. One unit of PON1 activity is defined as 1 *μ*mol of 4-nitrophenol formed per minute under the given conditions.

Lp-PLA_2_ was assessed by using 2-thio PAF as substrate, which is hydrolyzed by the enzyme in sn-2 position [31]. The consequent formation of free thiols was detected by Ellman's procedure. The reaction was started by adding sample to the reaction mixture containing buffer (100 mM Tris, 0.1 mmol/l ethylene glycol-bis(2-aminoethylether)-N,N,N′,N′-tetra-acetic acid (EGTA), pH = 7.2), 0.5 mmol/l DTNB, and 0.2 mmol/l 2-thio PAF (Cayman Chemical, Ann Arbor, Michigan, USA) in each well. A molar extinction coefficient of 13,600 M^−1^ cm^−1^ was used for the enzyme activity calculation, expressed in unit per liter.

Hydroperoxides (HY) were assessed by colorimetric assay based on the reaction between these lipid peroxidation by-products and N,N-diethyl-para-phenylendiamine from Sigma-Aldrich (St. Louis, MO, USA) [32, 33]. Briefly, serum or standard (H_2_O_2_) was added to a solution containing 190 *μ*l of acetate buffer (pH 4.8) and 5 *μ*l of chromogen (0.0028 M). The solution was incubated at 37°C and then read for optical density after 1 and 4 minutes. The concentration of hydroperoxides was obtained by the average ΔA505/min and expressed as U Carr, where 1 UC corresponds to 0.023 mM of H_2_O_2_.

AOPP determination was based on the spectrophotometric detection according to Capeillère-Blandin [34]. Concentrations of AOPP, determined in reference to the calibration curve, were expressed in *μ*mol/l.

Ceruloplasmin concentration was determined by competitive enzyme-linked immunosorbent assay (ELISA) according to the manufacturer's protocol (AssayMax Human Ceruloplasmin ELISA kit EC4001–1, Assaypro LLC, St. Charles, MO, USA; intra-assay CV and interassay CV were 3.6 and 9.1%, resp.) as previously described [35]. Serum TRAIL levels were analyzed in duplicate by using a specific ELISA kit (R&D Systems, Minneapolis, MN; intra-assay CV and interassay CV were 3.9 and 6%, resp.) in agreement with the manufacturer's instructions as previously described [36] while serum levels of hs-CRP, total cholesterol, LDL cholesterol (LDL-C), HDL cholesterol (HDL-C), and triglycerides were measured by routine laboratory methods.

### 2.3. Cell Treatments and RT-PCR Analysis

Peripheral blood mononuclear cell (PBMC) from healthy volunteers was isolated and resuspended in RPMI 1640 culture medium supplemented with GlutaMAX I, 25 mM HEPES buffer, and 10% fetal bovine serum (GIBCO BRL, Invitrogen). Cells were treated with recombinant TNF-*α* (R&D Systems, Minneapolis, MN) at the optimal concentration of 50 ng/ml determined in preliminary dose-response experiments used alone or associated with 0.01–0.1 *μ*g/ml of recombinant TRAIL (rhTRAIL) prepared as previously described [37]. In the combined treatments, rhTRAIL was added to the cells at the indicated concentrations 1 hour before treatment with TNF-*α*. Cell viability was monitored by means of trypan blue dye exclusion.

Total RNA was extracted by using the RNeasy Mini kit (Qiagen) by following the manufacturer's instruction and as previously described [38]. Briefly, after genomic DNA removal with RNase-Free DNase Set (Qiagen), 200 ng of RNA was retrotranscribed and amplified with the Express 1step SS qRT-PCR Universal (Life Technologies) with the TaqMan assay technology. The POLR2A was used as housekeeping gene. The TaqMan assays used were Hs00236810 (for ceruloplasmin) and Hs00172187_m1 (for POLR2A).

### 2.4. Statistical Analysis

Continuous variables were first analyzed for the normal distribution by the Shapiro-Wilkinson test. Simple correlation analyses were performed using Pearson's and Spearman's tests for normally and nonnormally distributed variables, respectively. Since the distribution of some variables was normal upon base-10 logarithm transformation, we used the log values for correlation analyses. Multiple regression analysis was performed to determine whether the emerged associations were independent from potential confounders. Two-tailed probability values < 0.05 were considered statistically significant.

## 3. Results

### 3.1. Levels of Circulating Oxidative Stress Markers and TRAIL in the Whole Cohort of Subjects

The main anthropometric and biochemical serum parameters, including the levels of markers of oxidative damage to lipids (hydroperoxides) and proteins (AOPP), ceruloplasmin, Lp-PLA_2_, and paraoxonase as well as TRAIL serum levels of the cohort of subjects (78% women) enrolled in the present study, are summarized in Table 1. As displayed (Table 1), the prevalence of smokers and diabetic subjects was very low (less than 13% in both cases), while the 68% of men and the 19% of women were presenting hypertension.

### 3.2. Differential Association between Serum TRAIL Levels and Circulating Levels of Oxidative Stress Markers

The first step of our analysis was focused on revealing possible association between the circulating enzymes with antioxidant proprieties (Lp-PLA2, ceruloplasmin, and PON1) and TRAIL. Due to the well-known influence of age and gender to oxidative stress (OxS) processes, we adjusted the analyses for these two covariates. Among the parameters considered, only ceruloplasmin showed an age-gender independent and inverse association with TRAIL (*p* < 0.001, *R* ^2^ = 0.142) (Table 2). Of note, looking for potential correlations between TRAIL and markers of oxidative damage, we found that only hydroperoxides were significantly correlated to TRAIL (*p* < 0.001). However, this association was weak and was not confirmed after adjusting for age and gender.

The second phase of the statistical analysis aimed to verify the independence of the emerged association between TRAIL and ceruloplasmin through a multiple regression test including as covariates of hypertension, diabetes, and smoking habit besides age and gender. As shown in Table 3, the multivariate analysis showed that the targeted association was not affected by the additional potential cofounding factors (*p* < 0.001, contribution to the *R* ^2^ of the multiadjusted model = 0.171). Finally, since diabetes represents a factor that affects the levels of circulating TRAIL, we performed a further analysis of the correlation between TRAIL and ceruloplasmin in the subgroup of samples without diabetic subjects that confirmed the strength and independence of the association (Supplementary Figure 1).

### 3.3. Inverse Correlation between the Circulating Levels of TRAIL and Ceruloplasmin

**Figure 1:**
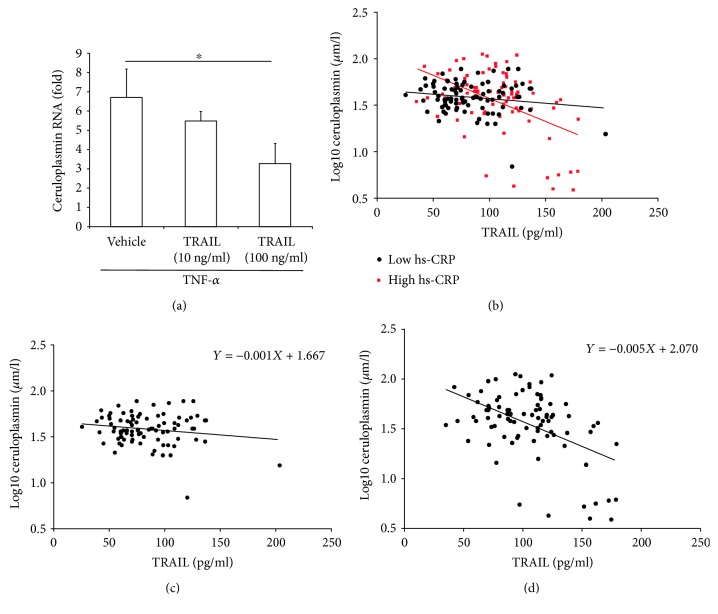
Role of inflammation in the correlation between TRAIL and ceruloplasmin. In (a), PBMC cultures were exposed to TNF-*α* (50 ng/ml) alone or in combination with recombinant human TRAIL (used at the indicated concentrations) for 24 hours and analyzed for ceruloplasmin mRNA levels by quantitative RT-PCR. Data are reported as means ± SD of results obtained in total PBMCs expressed as fold of induction with respect to untreated cultures (set to 1). ^∗^ *p* < 0.05 with respect to TNF-*α*-treated cultures. In (b)-(d), inverse correlation between TRAIL and ceruloplasmin circulating levels in the whole sample. Scatter plot shows the relationship between the serum levels of TRAIL and ceruloplasmin after division of the whole population in two groups according to the hs-CRP median value (hs-CRP: 5.1 nmol/l), red square data points = samples with high hs-CRP levels (>5.1 nmol/l) and full black circle data points = samples with low hs-CRP levels (≤5.1 nmol/l) (b). Single scatter plots and regression equations of the correlation between TRAIL and ceruloplasmin levels within the low hs-CRP and high hs-CRP groups (c and d, resp.).

To assess whether TRAIL might be directly involved in the downregulation of ceruloplasmin, we carried out *in vitro* experiments by using PBMCs, which are known to express ceruloplasmin and are thought to significantly contribute to the release of ceruloplasmin in the systemic circulation [39, 40]. Since circulating immune cells are potential targets of TRAIL and activated monocytes are a major source of ceruloplasmin [40, 41], we checked the putative effect of TRAIL on ceruloplasmin expression on PBMCs under TNF-*α* stimulation. As shown in Figure 1(a), cell treatment with recombinant TRAIL induced a significant downmodulation of the TNF-*α*-induced ceruloplasmin mRNA levels in a dose-dependent manner. The evidence that TRAIL had negligible effects on cell viability ruled out the possibility that ceruloplasmin downmodulation was a mere indirect consequence of the cytotoxic effect of TRAIL. On the contrary, no effect was detected in the absence of an inflammatory milieu.

To confirm *in vivo* the potential role of inflammation in the link between TRAIL and ceruloplasmin, we stratified the population sample in two subsamples using the median level of hs-CRP, the mostly used marker of systemic inflammation, as discriminating threshold (median value = 16.18 nmol/l) (Figure 1(b)). As shown, in the subgroup characterized by higher hs-CRP (named as high hs-CRP), the correlation between the two variables was strong and significant (Figure 1(d); *r* = −0.508, *p* < 0.001, *R* ^2^ = 0.260). On the contrary, within the low hs-CRP subgroup, TRAIL and ceruloplasmin were not correlated to each other (Figure 1(c); *r* = −0.168, *p* = 0.117, *R* ^2^ = 0.028). As depicted by the figure, the difference between TRAIL versus ceruloplasmin interplay in the two subsamples was evident also considering the slope of the regression lines, with that of high hs-CRP markedly steeper than that of the others (−0.005 versus −0.001). Noteworthy, the association found in high hs-CRP was independent of age, gender, smoking, hypertension, and diabetes. (Supplementary Table 1, *β* = 0.443, *p* < 0.001, *R* ^2^ = 0.316). Finally, the above reported correlation results did not significantly change using the same hs-CRP threshold used to discriminate elderly individuals in InCHIANTI study (i.e., 28 mmol/l) [42].

## 4. Discussion

Results of the present study indicated that high circulating levels of TRAIL are associated with low ceruloplasmin levels and are independent of potential confounding factors, such as age, sex, and comorbidities. Moreover, *in vitro* studies confirmed that the inverse association between TRAIL levels and ceruloplasmin levels was evident only in the presence of an inflammatory milieu, which indicated that a signaling axis involves TRAIL and ceruloplasmin that are linked via inflammation.

TRAIL is generally recognized as a pleiotropic protein that exerts protective effects within the vasculature, although contrasting results have been reported and its mechanisms of action still remain unclear. TRAIL can act directly on the vasculature by binding to receptors that are also localized in the endothelium and vascular smooth muscle cells. TRAIL can also act indirectly by modulating the expression of various pro/anti-inflammatory mediators. Proposed targets of these modulation pathways are adhesion molecules, such as ICAM-1, E-selectin, VCAM, the vasoconstrictor endothelin-1 [2], and adipokines, such as resistin and lipocalin 2/ngal [43]. More recently, redox/redox-sensitive signaling pathways and OxS have been suggested to be involved in mediating the effects of TRAIL in the context of several diseases, particularly CVD.

Ceruloplasmin is an inflammatory, acute-phase plasma protein that is expressed by liver hepatocytes and activated monocyte/macrophages. The main physiological function of ceruloplasmin is the transport and delivery of copper to tissues [39]. In addition, ceruloplasmin appears to exert protective effects against OxS because of its ability to isolate copper from the plasmatic environment and its well-described ferroxidase activity. Indeed, free reduced iron (Fe^2+^) and copper (Cu^+^) can react with organic hydroperoxides (by-products of lipid peroxidation) or hydrogen peroxide (abundantly produced during normal cell metabolism) to form the highly toxic alcoxyl and hydroxyl radicals, respectively. Ferroxidase has also been implicated in iron homeostasis because it catalyzes the oxidation of free iron to promote iron incorporation into apotransferrin or the Fe storage protein ferritin [18, 44]. Notably, iron accumulates in the brains and livers of patients with absent/dysfunctional ceruloplasmin and ceruloplasmin knockout mice, which in turn leads to diabetes and dementia [45]. However, the beneficial role of ceruloplasmin contradicts with the well-documented association between high circulating levels of ceruloplasmin and CVDs. High ceruloplasmin/copper levels have been reported in patients with atherosclerosis and myocardial infraction. Recently, Tang et al. showed that ceruloplasmin is a strong and independent predictor of long-term adverse cardiac outcomes in stable cardiac patients [46]. High ceruloplasmin levels have been associated with type I and type II diabetes, metabolic syndrome, and other well-established CVD risk factors, suggesting that it has a more predominant role in signaling pathways than in exerting antioxidant properties. Considering that inflammation is an important determinant of CVDs and that ceruloplasmin is an inflammatory-sensitive plasma protein, inflammation could, at least in part, explain the observed discrepancy in the results. Indeed, a compelling body of evidence supports the idea that the mechanistic role of ceruloplasmin in vascular diseases is independent of its function as an acute-phase protein [18]. Furthermore, studies have demonstrated that copper has a central role in mediating the pathological effects of ceruloplasmin and that OxS is likely to be the primarily responsible for the release of the prooxidant copper in the blood and at the blood plasma interface. Similar to other proteins, such as PON1, ceruloplasmin is highly susceptible to ROS, and its induced oxidative modification leads to the loss of structural integrity of the protein, release of bound copper, and impaired ferroxidase activity [47, 48]. Overall, the putative shift from its antioxidant (protective) to prooxidant (vasculopathic) role could be dependent on conditions of pronounced redox imbalance, such as in diabetes and generally in the presence of chronic inflammation [49]. At the same time, in the presence of low-grade inflammation, elevated ceruloplasmin concentrations might not be detrimental to the vasculature [18, 50].

Our results demonstrated the inhibitory effects of TRAIL on ceruloplasmin expression, thereby contributing new insights on a TRAIL-regulated factor that can influence physiopathological processes in the vascular system. In addition to hepatocytes, ceruloplasmin is expressed by circulating monocytes, resident macrophages, and lymphocytes [39]. Furthermore, inflammatory mediators, such as TNF-*α* and interleukin-6, can induce ceruloplasmin expression in these cells [40]. Taken together, our findings suggested that TRAIL can downregulate the expression of ceruloplasmin in circulating immune cells only in the presence of an evident inflammatory state. Intriguingly, consistent with the observed association between inflammation and OxS, the subsample of patients that showed high hs-CRP levels also had significantly higher hydroperoxide levels compared to the remaining patients (354 ± 96 versus 291 ± 96 UC, mean ± SD, *p* = 0.001; data not shown). Thus, these patients are more likely to have higher vasculopathic ceruloplasmin/vasculoprotective ceruloplasmin ratios. We are nonetheless aware about some important limitations of our study that must be acknowledged. These are mainly related to the lacking of specific clinical/epidemiological information (e.g., BMI of subjects and presence of specific additional pharmacological treatments such as bone protection treatments) that might influence the circulating levels of TRAIL and/or those of its decoy receptor OPG and therefore partially affecting the reliability and the clinical significance of the associations. Nevertheless, we believe that our findings demonstrating a link between TRAIL and ceruloplasmin through inflammation may provide the proof of concept for longitudinal investigations. To our knowledge, our study is the first to investigate the relationship between TRAIL and OxS markers, thereby providing new insights on the potential pathways mediating the vasoprotective effects of TRAIL. Further studies under different pathological contexts are required to verify the hypothesis that TRAIL exerts its protective effects by blocking ceruloplasmin activity, which could in turn provide the basis for the development of therapeutic strategies.

## Figures and Tables

**Table 1:** Principal characteristics of the subjects.

Demographic and clinical parameters	Value
Number of subjects, *n*	209
Age, years	64 ± 14
Gender (W/M), *n*	163/46
Smoking, *n* (%)	19 (8)
Hypertension, *n* (%)	62 (30)
Diabetes, *n* (%)	18 (9)

Serum parameters	

PON1 (U/l)	95 (48–167)
Lp-PLA_2_ (U/l)	16.4 ± 4.7
Ceruloplasmin (*μ*mol/l)	2.9 (2.2–3.8)
Hydroperoxides (UC)	322 ± 112
AOPP (*μ*mol/l)	70 (57–85)
hs-CRP (nmol/l)	51 (16–121)
TRAIL (pg/ml)	93 ± 36

Data are expressed as % within the group for categorical variables and number of subjects in brackets, mean ± standard deviation for normally distributed continuous variables, and median (interquartile range) for nonnormally distributed continuous variables. PON1: paraoxonase 1; Lp-PLA_2_: lipoprotein phospholipase A2; AOPP: advanced oxidation protein products; hs-CRP: high-sensitivity C-reactive protein.

**Table 2:** Simple and partial (age- and gender-adjusted) correlation coefficients for the association between TRAIL and redox-related markers.

Redox-related markers	Correlation coefficient (*r*)	*p*	Adjusted correlation coefficient (*r*_p_)	*p*
Lp-PLA_2_	**−0.194**	**0.020**	**−**0.155	0.07
Ceruloplasmin	**−0.407**	**<0.001**	**−0.431**	**<0.001**
PON1	0.071	0.420	−0.061	0.601
Hydroperoxides	**0.291**	**<0.001**	0.107	0.152
AOPP	0.130	0.106	0.155	0.071

Lp-PLA_2_: lipoprotein phospholipase A_2_; PON1: paraoxonase 1; AOPP: advanced oxidation protein products.

**Table 3:** Multiple regression analysis of the association between TRAIL and ceruloplasmin.

Outcome variables	Explanatory variable	*B*	Standard error	*β* (*p*)	Contribute to outcome variance	*R* ^2^^§^
Ceruloplasmin	TRAIL	−0.004	0.001	−0.469 (<0.001)	0.171^#^	0.203

Multiple regression models include age, gender, hypertension, diabetes, and smoking status. *B*: unstandardized regression coefficient; *β*: standardized regression coefficient. ^#^The squared semipartial correlation coefficient accounts for the proportion of variance in the dependent variable that is explained by the covariate. ^§^The overall model.

## Data Availability

The data used to support the findings of this study are available from the corresponding author upon request.

## Supplementary Materials

Supplementary Materials Supplementary Figure 1. Association between TRAIL and Log10 ceruloplasmin in the whole sample (including diabetic subjects) and in subsample including only subjects without diabetes. In A, scatter plot showing the association in the whole sample (*n* = 209) with data points of the subjects with diabetes (*n* = 18) highlighted in red (square). Pearson's correlation coefficient: *r* = −0.407 (*p* < 0.001). In B, scatter plot showing the association in the subsample including subjects without diabetes (*n* = 191). Pearson's correlation coefficient *r* = −0.421 (*p* < 0.001). Supplementary Table 1. Multiple regression analysis of the association between TRAIL and ceruloplasmin in subsamples stratified according to hs-CRP median value.
