# Supplementary material for: TRAIL and Ceruloplasmin Inverse Correlation as a Representative Crosstalk between Inflammation and Oxidative Stress
Source: Mediators Inflamm. 2018 Jul 26;2018:9629537. doi: 10.1155/2018/9629537 (PMC6083483; doi:10.1155/2018/9629537)
Supplement: Supplementary Materials — Supplementary Figure 1. Association between TRAIL and Log10 ceruloplasmin in the whole sample (including diabetic subjects) and in subsample including only subjects without diabetes. In A, scatter plot showing the association in the whole sample (n = 209) with data points of the subjects with diabetes (n = 18) highlighted in red (square). Pearson's correlation coefficient: r = −0.407 (p < 0.001). In B, scatter plot showing the association in the subsample including subjects without diabetes (n = 191). Pearson's correlation coefficient r = −0.421 (p < 0.001). Supplementary Table 1. Multiple regression analysis of the association between TRAIL and ceruloplasmin in subsamples stratified according to hs-CRP median value. [file 9629537.f1.pdf]

Supplementary Figure 1

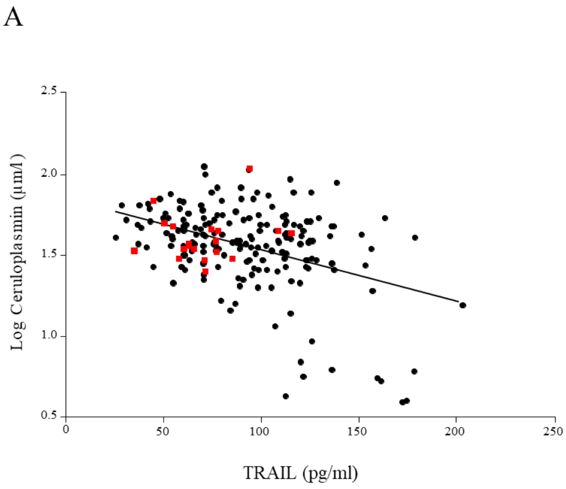

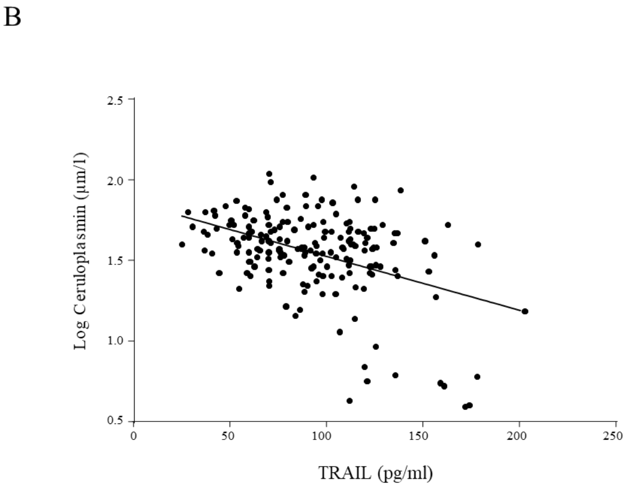

Supplementary Table 1. Multiple regression analysis of the association between TRAIL and ceruloplasmin in subsamples stratified according to Hs-CRP median value

**Hs.CRP levels**	*B*	*Standard error*	*β*	*Contribute to outcome variance*	$R^{2§}$
**LOW (≤5.1nmol/l)**	-0.001	0.001	-0.213 (0.085)	0.035#	0.047
**HIGH (>5.1nmol/l)**	-0.004	0.001	-0.443 (<0.001)	0.140#	0.316

Multiple regression models include, age, gender, hypertension, diabetes and smoking status; B= unstandardized regression coefficient; β= standardized regression coefficient; # The squared semi-partial correlation coefficient accounts for the proportion of variance in the dependent variable that is explained by the covariate; § referred to the overall model
